# Supplementary material for: TIM-3 Expression on Dendritic Cells in Colorectal Cancer
Source: Cancers (Basel). 2024 May 15;16(10):1888. doi: 10.3390/cancers16101888 (PMC11120027; doi:10.3390/cancers16101888)

TIM-3 expression on dendritic cells in colorectal cancer

Mei Sakuma, Masanori Katagata, Hirokazu Okayama, Shotaro Nakajima, Katsuharu Saito, Takahiro Sato, Satoshi Fukai, Hideaki Tsumuraya, Hisashi Onozawa, Wataru Sakamoto, Motonobu Saito, Zenichiro Saze, Tomoyuki Momma, Kosaku Mimura and Koji Kono.

Supplemental Figure S1

A

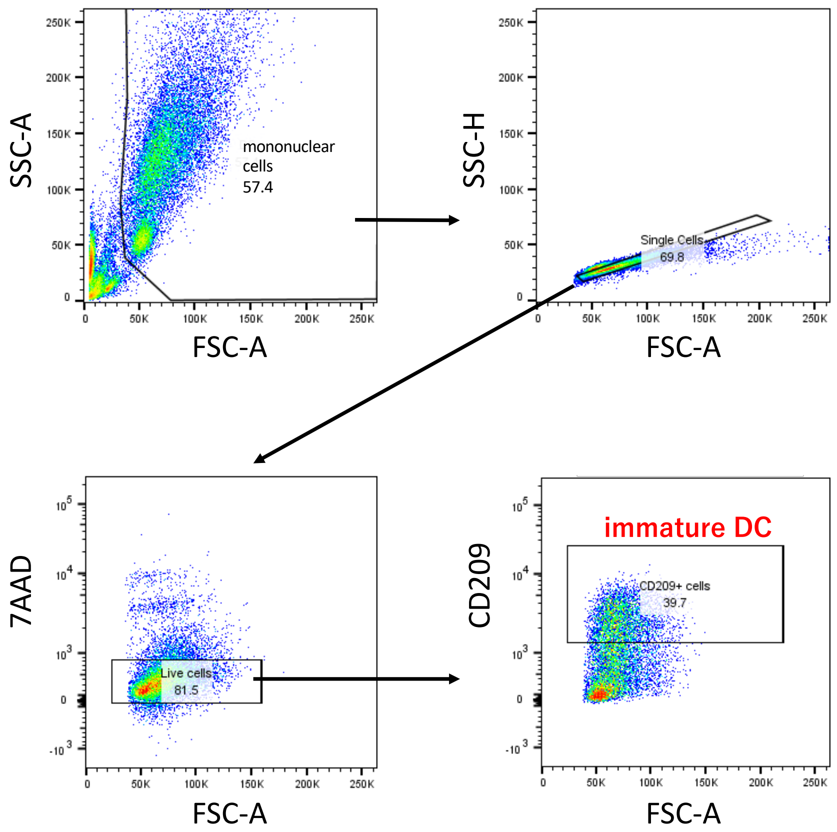

B

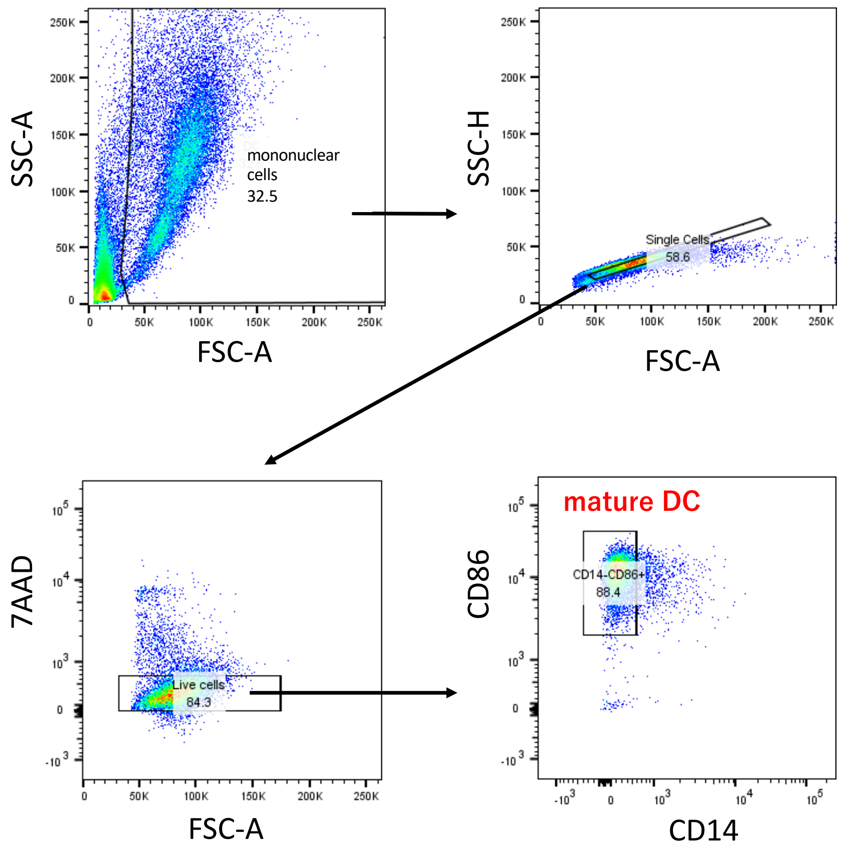

TIM-3 expression on dendritic cells in colorectal cancer

Mei Sakuma, Masanori Katagata, Hirokazu Okayama, Shotaro Nakajima, Katsuharu Saito, Takahiro Sato, Satoshi Fukai, Hideaki Tsumuraya, Hisashi Onozawa, Wataru Sakamoto, Motonobu Saito, Zenichiro Saze, Tomoyuki Momma, Kosaku Mimura and Koji Kono.

Supplemental Figure S2

A

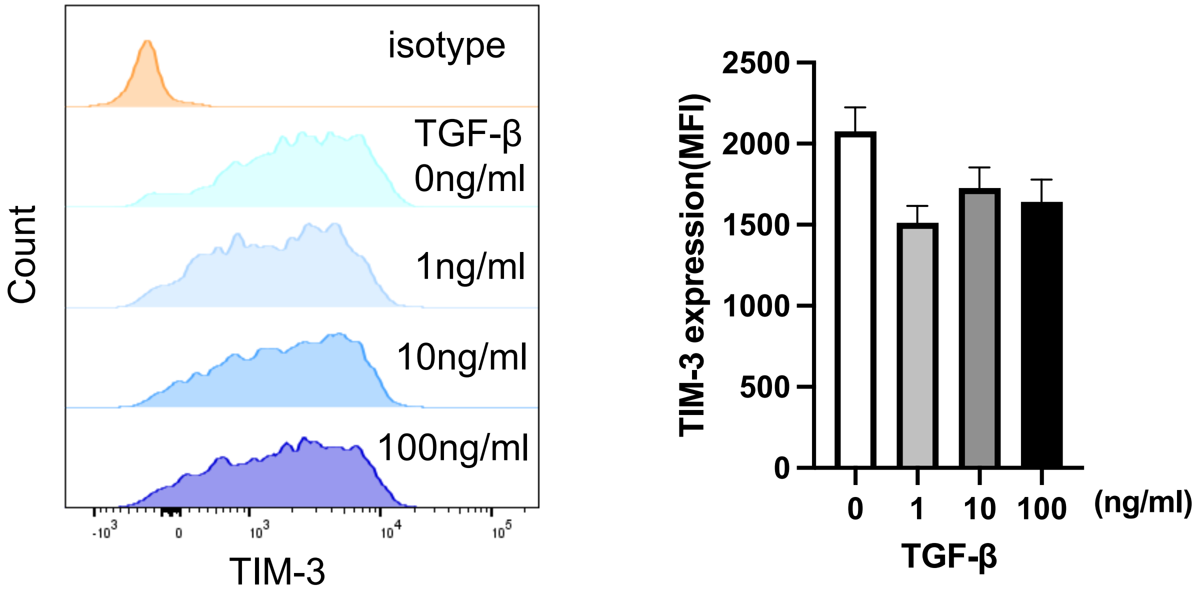

B

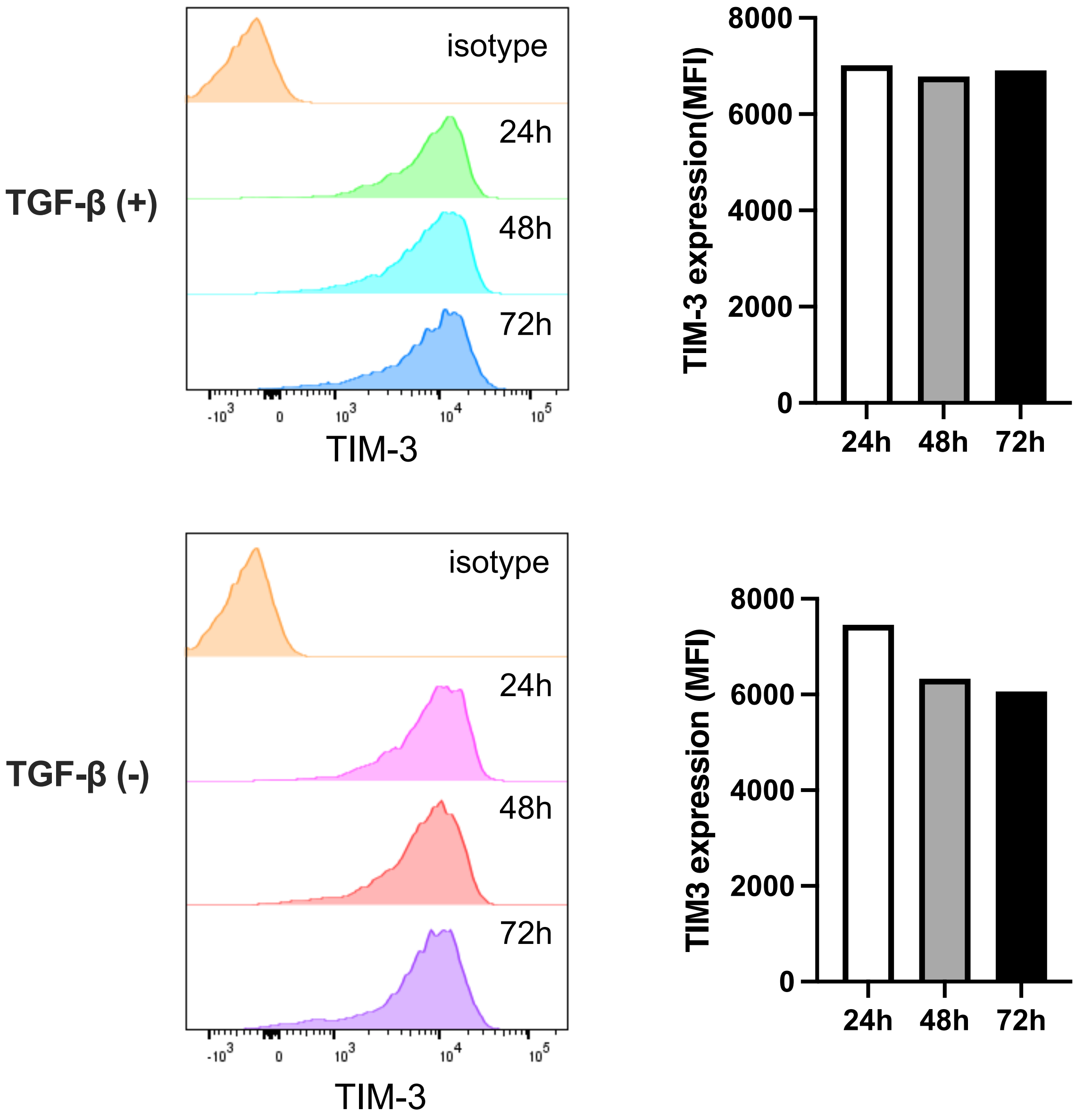

TIM-3 expression on dendritic cells in colorectal cancer

Mei Sakuma, Masanori Katagata, Hirokazu Okayama, Shotaro Nakajima, Katsuharu Saito, Takahiro Sato, Satoshi Fukai, Hideaki Tsumuraya, Hisashi Onozawa, Wataru Sakamoto, Motonobu Saito, Zenichiro Saze, Tomoyuki Momma, Kosaku Mimura and Koji Kono.

Supplemental Figure S3

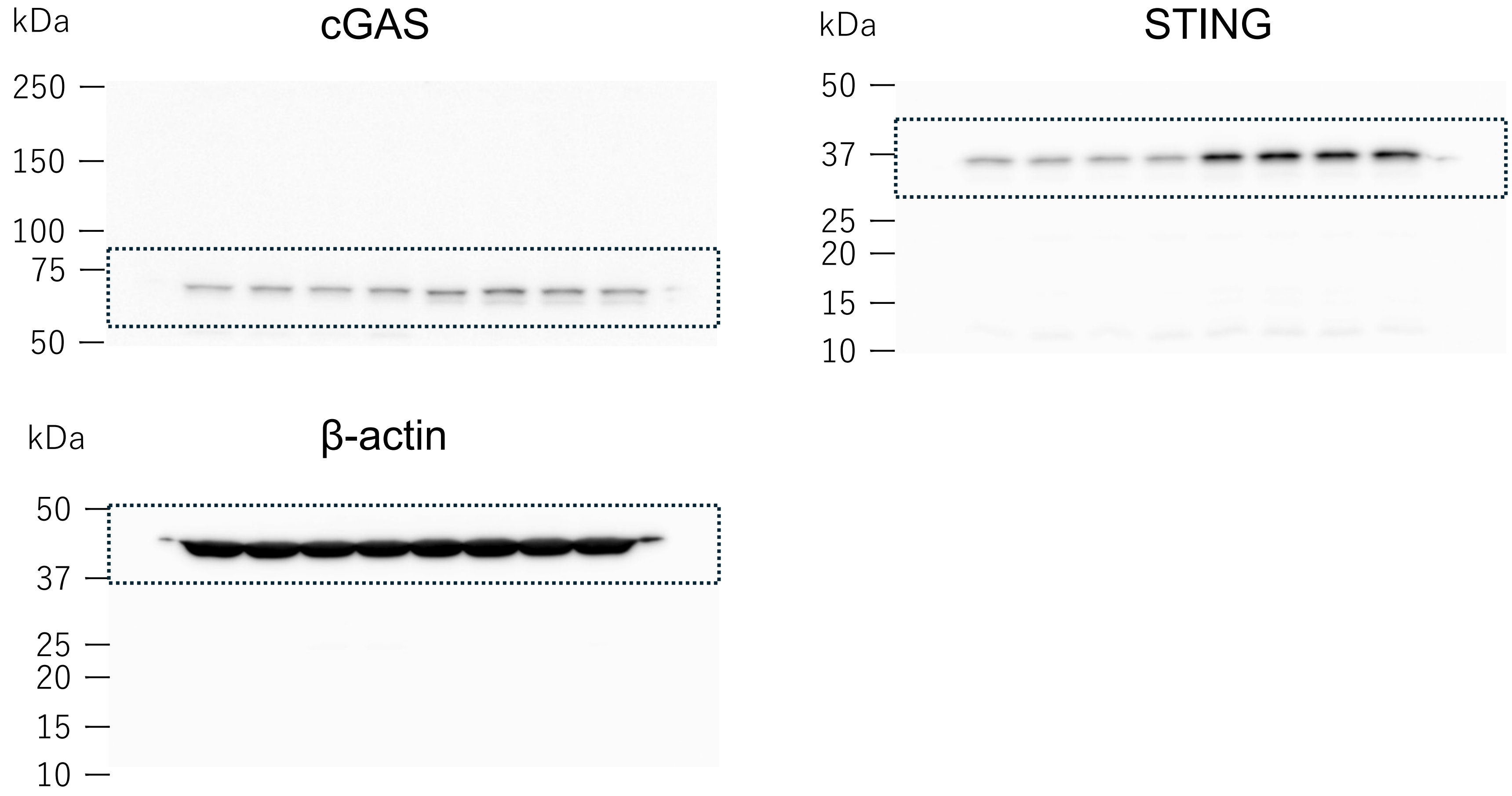

Supplement: Supplementary file 1 [file cancers-16-01888-s001.zip › cancers-2960081-supplementary.pdf]
